# Unraveling the role of early coeliac disease diagnosis in the risk of developing immune-mediated renal diseases

**DOI:** 10.1186/s12876-025-03705-5

**Published:** 2025-03-03

**Authors:** Francesco De Luca, Staffan Nilsson, Katarina Truvé, Hans-Georg Kuhn, Katarina Ejeskär, Börje Haraldsson, Åsa Torinsson Naluai

**Affiliations:** 1 Department of Laboratory Medicine, Institute of Biomedicine, Sahlgrenska Academy at the University of Gothenburg, Gothenburg, Sweden; 2 Core Facilities, Sahlgrenska Academy at the University of Gothenburg, Gothenburg, Sweden; 3 Department of Clinical Neuroscience, Institute of Neuroscience and Physiology, Sahlgrenska Academy at the University of Gothenburg, Gothenburg, Sweden; 4 Translational Medicine, DHEAR, Institute of Health Sciences, Skövde University, Skövde, Sweden

**Keywords:** Coeliac disease, Age at celiac disease diagnosis, Immune-mediated renal diseases, Risk of development

## Abstract

**Background:**

coeliac disease (CD) is an inflammatory condition of the small intestine caused by immunological intolerance towards dietary gluten. Associations between CD and other autoimmune disorders have been extensively reported. However, the risk in CD patients of developing immune-mediated renal diseases (IMRDs) as a function of the duration of exposure to gluten remains uncharacterized.

**Methods:**

we used data from the Swedish national patient register to retrospectively construct two subcohorts of CD patients by either years before or after CD diagnosis, matched by sex and age to reference individuals (ratio 1:6). Adopting cox regressions, we assessed the risk in CD to develop IMRDs.

**Results:**

we found that unrecognized CD patients had a higher risk to develop the majority of the IMRDs here investigated compared with matched reference individuals. Following a CD diagnosis, the risk was reduced in eight of the twelve IMRDs. Furthermore, if patients were diagnosed with CD earlier in childhood they showed less or no increased risk to develop IMRDs compared with reference individuals. CD patients diagnosed by the age of 15 had an overall 12% increased risk of developing any IMRD, (HR: 1.12; CI = 1.02, 1.24; *p* < 0.02), as those with a CD diagnosis between 16 and 30 years of age had a 60% increased risk of developing IMRD (HR: 1.61; CI = 1.36, 1.91; *p* < 0.001).

**Conclusions:**

Our data show that individuals diagnosed with CD at an earlier age have a lower risk of developing immune-mediated kidney conditions.

**Supplementary Information:**

The online version contains supplementary material available at 10.1186/s12876-025-03705-5.

## Background

Coeliac disease (CD) is an inflammatory condition of the small intestine where individuals with genetic predisposition develop, under triggering events, immunological intolerance towards dietary gluten [1]. Sensitivity to dietary gluten does not necessarily imply the development of significant symptoms. If symptoms arise, they typically begin to appear in childhood although CD can manifest at any age [2]. The diversity of manifestations related to CD is causing a general delay in diagnosis [3]. A significant number of seemingly healthy individuals are subclinical and suffer from silent CD. These undiagnosed CD individuals coexist with non-classic signs and long-term complications [4, 5]. However, initiation of a strict gluten-free diet following diagnosis usually leads to symptom improvement [6]. The general adherence to a gluten-free diet is good. For instance, a long-term follow-up study from 2014 showed that 96.8% of Swedish children with CD tried to keep a strict gluten-free diet [7].

In CD, T-cells are activated by gliadin and glutenin peptides; the two main components of gluten [8]. T-cell activation following immunological presentation of gluten peptides differs between children and adults. T-cells from pediatric CD patients respond to and are activated by glutenin peptides with minor immunogenic capacities [9]. In contrast, the interaction between T-cells and deaminated gliadin peptides in adults leads to enhanced immune response [10, 11]. This suggests that longstanding inflammation, possibly due to late CD diagnosis, may promote potent T-cell activation. Intriguingly, a previous study found that CD diagnosed early in childhood associated with fewer co-occurring autoimmune diseases [12].

Tissue transglutaminase (tTG) is known to catalyze deamination of gliadins [13]. Deaminated gliadin peptides have been found to bind to and complex with components of intestinal matrix collagens. This suggests that cross-reactivity-mediated expansion of the humoral response directed against collagens may occur in the CD population [14]. It is believed that this process may contribute to the development of collagen-associated inflammatory and autoimmune disorders in CD [15]. Among several other organs, individuals with CD have been shown to be at risk to develop diseases of the kidney [16].

There are known associations between CD and an increased risk of developing renal diseases. Studies have shown that patients with CD are at higher risk for conditions such as end-stage renal disease [17], immunoglobulin A nephropathy, and chronic glomerulonephritis [18]. Additionally, children with type 1 diabetes who follow a gluten-free diet due to a CD diagnosis have been shown to have a reduced risk of renal disease [19]. Conversely, non-adherence to a gluten-free diet in children with both type 1 diabetes and CD has been linked to an elevated albumin excretion rate [20]. Moreover, gluten-free diet, even without CD, has shown to have anti-inflammatory effects in children with steroid-resistant nephrotic syndrome [21].

Despite this potential benefit of gluten-free diet [22], the risk of developing autoimmune renal diseases in CD as a function of age at CD diagnosis has not been investigated.

In this study we want to investigate if early CD diagnosis reduces the risk of developing CD-associated immune-mediated renal diseases, here mentioned as IMRDs. To test our hypothesis, we conducted a registry-based retrospective cohort study in which we used data from the registers at the Swedish National Board of Health and Welfare to examine individuals with CD and IMRDs. We focused on autoimmune diseases of the kidney both at the glomerular and tubular level leading to nephrotic syndrome, nephritic syndrome, rapidly progressive glomerulonephritis and tubulointerstitial nephritis [23]. These are clinical conditions that can affect individuals at any age and occur with a multitude of symptoms such as proteinuria, low serum albumin, oedema, oliguria, hyperlipidemia, hematuria and high blood pressure [24]. It is important to identify any significant variation of the risk of renal autoimmune disorders in the CD population, because these diseases are associated with increased morbidity and mortality [25].

## Methods

### Study populations and definition of CD and IMRDs

The dataset was prepared and pseudonymized by the Swedish government agency of statistics. All participants were identified using their personal identity number, which is a 10-digit number given to all Swedish residents at birth or immigration and used as personal identifier in medical records [26]. Medical data were obtained from the Swedish National Board of Health and Welfare and consisted of an extract from inpatient and outpatient medical registers used for the investigation of chronic diseases with inflammatory components between 1964 and 2017. The dataset included individuals who were themselves affected or who had parents, children or siblings affected by CD or diabetes. The International classifications of disease (ICD) codes used to identify these patients were 579A, 648A, 648W, 286, 269, 250, 260, K90.0 and E10-E14 O24. In total, approximately 3.3 million individuals or 30% of the total Swedish population were included.

Following exclusion of participants with diabetes, six sex- and age-matched reference individuals (RI) were randomly selected for each CD participant. There were 56 240 CD participants with 306 942 matched RI (Table 2). We then excluded those CD patients that died the same year they were diagnosed with CD and for who CD diagnosis occurred after 75 years of age. We further excluded RI that died the same year or before as their matching CD individual acquired CD diagnosis. This resulted in a main CD cohort of 42 355 participants, each with six matched RI (Table 2). Finally, we assessed medical records of CD patients and RI for diagnoses of IMRDs (outcomes) using relevant ICD codes. ICD N00-N06 cover all glomerular diseases except the hereditary forms. The code N08 was also included since it is a code that is used for the glomerular disease when the main disease is classified with another code. N10-12 covers the tubulointerstitial nephritis and the inflammatory forms of this disease. We also added M31, M35 and M32, which are vasculitis, Sjögren’s syndrome and SLE with inflammatory kidney involvement respectively. In addition, D69.0 (Henoch-Schönlein purpura) and D59 (Acquired hemolytic anemia) have inflammatory components involving the kidney and were therefore also included (Table 1).

**Table 1:** International Classification of Diseases (ICD) 10, ICD-9, ICD-8 and ICD-7 codes were used to identify patients diagnosed with immune-mediated renal diseases (IMRDs)

Disease classification	ICD-10 (> 1997)	ICD-9 (1987–1997)	ICD-8 (1968–1986)	ICD-7 (1964–1968)
Acute nephritic syndrome ***(ANS)***	N00	580 [A,B,C,D,E,W,X]	580	590
Rapidly progressive nephritic syndrome ***(RPNS)***	N01	580 [A,B,C,D,E,W,X]	580	
Recurrent and persistent hematuria ***(RPE)****,* Gross hematuria	N02, R31	599.7[H]	789.3	
Chronic nephritic syndrome ***(CNS)***	N03	582 [A,B,C,D,E,W,X]	582	592
Nephrotic syndrome ***(NS)***	N04	581 [A,B,C,D,W,X]	581	591
Unspecific nephritis and nephropathy ***(UNN) &*** Isolated proteinuria with specified morphological lesion ***&*** Glomerular disorders in diseases classified elsewhere	N05, N06 & N08	583 [A,B,C,D,W,X]	583	593
Tubulointerstitial nephritis ***(TIN)***	N10-12	590 [A,B,C,D,W,X], 593.3, 593.4	590	
Glomerulonephritis due to Henoch-Schönlein purpura ***(GHSP)***	D69	287.0, [A]	287.0	296.14
Acquired hemolytic anemia ***(AHA)***	D59	283.0, [A]	283.0	292.0, 292.1
Vasculitis & Wegner’s granuloma ***(WG)***	M31	446 [A,B,C,D,E, W,X]	446	
Systemic lupus erythematosus ***(SLE)***	M32.14	710.0 [A]	734.1	705.4
Sjögren’s syndrome ***(SS)***	M35.0	710.2 [C]	734.9	374.06

### Data management and statistical analyses

Cox regressions and the Kaplan–Meier curves were adopted to estimate the hazard ratio (HR) and plot cumulative events respectively to show the risk for CD individuals and their matched RI to develop IMRDs over time. The main package used in R (version 4.2.2) was Survminer. Time to event data analyses were conducted in two subcohorts, distinguishing between events before and after a CD diagnosis. In the subcohort “Before CD diagnosis”, age at CD diagnosis was considered the end of follow-up. In the subcohort “After CD diagnosis”, follow-up started the year after age at CD diagnosis and ended at age at IMRDs diagnosis, age at death, or age at 31st of December 2016 (whichever occurred first). A *p*-value < 0.05 was considered statistically significant. We used Fishers exact test to evaluate the difference in the groups before and after CD diagnosis. We also evaluated the difference between two age groups of CD onset (0–15 and 16–30) using conditional logistic regression of the interaction between a diagnosis of CD and age group in predicting risk of IMRDs.

## Results

### Association between CD and IMRDs

In Table 2, the total number of individuals included in the analysis are shown.

**Table 2:** Number of all individuals, reference individuals (RI) and those diagnosed with Coeliac Disease (CD)

	**All individuals**	**Reference Individuals (RI)**	**CD**
All born in Sweden until 2017	3 213 176	3 156 936	56 240
Matched 6 RI to each CD case	358 099	306 942	51 157
Removed those who died before CD diagnosis	296 485	254 130	42 355

By means of cox regressions, we found associations between CD and ten of the disease groups here investigated (Table 3).

**Table 3:** Immune Mediated Renal Diseases (IMRDs) in those diagnosed with Coeliac Disease (CD) and in reference individuals (RI)

Disease (ICD10)	Total IMRD	Matched IMRD	Mean age of IMRD	n (%) in CD	*n* (%) in RI	HR (95% CI)
**ALL IMRDs**	**260 456**	**14 959**	**57.7**	**2 666 (6.29%)**	**12 293 (4.84%)**	1.33 (1.28, 1.39)***
AHA (D59)	3 705	212	62.0	72 (0.17%)	140 (0.06%)	3.15 (2.37, 4.19)***
ANS (N00)	3 601	214	35.9	37 (0.09%)	177 (0.07%)	1.26 (0.88, 1.79)^NS^
ANS & RPNS	4 245	271	38.9	47 (0.11%)	224 (0.09%)	1.27 (0.93, 1.74)^NS^
CNS (N03)	11 299	390	48.9	72 (0.17%)	318 (0.13%)	1.38 (1.07, 1.78)*
GHSP (D69)	21 975	1 852	54.5	391 (0.92%)	1 461 (0.57%)	1.63 (1.46, 1.82)***
NS (N04)	8 863	347	41.9	70 (0.17%)	277 (0.11%)	1.53 (1.18, 1.99)**
RPE (N02)	93 252	3 636	64.2	633 (1.49%)	3 003 (1.18%)	1.31 (1.20, 1.43)***
SLE (M32)	2 985	104	50.3	36 (0.08%)	68 (0.03%)	3.26 (2.17, 4.88)***
SS (M35)	9 280	506	52.4	190 (0.45%)	316 (0.12%)	3.67 (3.06, 4.39)***
TIN (N10-12)	103 949	8 400	55.4	1 374 (3.24%)	7 026 (2.76%)	1.19 (1.12, 1.26)***
UNN (N05,6&8)	28 020	781	57.8	157 (0.37%)	624 (0.25%)	1.54 (1.30, 1.84)***
WG (M31)	19 657	455	68.4	100 (0.24%)	355 (0.14%)	1.77 (1.42, 2.21)***

Those IMRDs that showed the strongest increased risk with CD were Sjögren’s syndrome with a three to four times increased risk (HR = 3.67; CI = 3.06, 4.39), systemic lupus erythematosus with over three-fold increased risk (HR = 3.26; CI = 2.17, 4.88) and acquired hemolytic anemia also with over three-fold increased risk (HR = 3.15; CI = 2.37, 4.19), as shown in Table 3.

### Risk of developing IMRDs by age at CD diagnosis

To further investigate whether the duration of exposure to gluten may affect the risk for patients with CD to develop IMRDs, we stratified our main CD cohort based on the time at which CD diagnosis occurred. This yielded two subcohorts: one before or during the same year as the CD diagnosis (end follow-up one year after CD diagnosis) and the other cohort starting one year after CD diagnosis.

There was a trend that patients were at increased risk to develop the majority of IMRDs before a diagnosis of CD was made compared with after a CD diagnosis was made. Acute nephritic syndrome (ANS) together with Rapidly progressive nephritic syndrome (RPNS), Recurrent and persistent hematuria (RPE) and Wegener’s granulomatosis (WG) showed a significant difference in risk before versus after a CD diagnosis (Table 4 and Figure S1).

**Table 4:** Immune Mediated Renal Diseases (IMRDs) in those diagnosed with Coeliac Disease (CD) and in Reference Individuals (RI) before vs after CD diagnosis

	**Before CD diagnosis**			**After CD diagnosis**			**Fisher test before vs after**
**IMRD**	**CD—RI [*n* (%)**^**a**^**]**	**HR (95%CI)**	***p-value***	**CD—RI [*n* (%)**^**a**^**]**	**HR (95%CI)**	***p-value***	**Estimate (95%CI)**
ALL	1 566 (59)—7 057(57)	1.35 (1.28, 1.43)	1.43E-25***	1 100 (41)—5 236 (43)	1.34 (1.25, 1.42)	2.6E-19***	0.95 (0.87, 1.03)
AHA	39 (54)—75 (54)	3.65 (2.45, 5.43)	1.68E-10 ***	33 (46)—65 (46)	2.74 (1.82, 4.13)	1.5E-06***	0.98 (0.53, 1.79)
ANS	30 (81)—121 (68)	1.47 (0.97, 2.22)	*0.063*	7 (19)—56 (32)	0.90 (0.45, 1.81)	*0.769*	0.51 (0.18, 1.27)
ANS & RPNS	37 (79)—141 (63)	1.55 (1.06, 2.27)	0.044*	10 (21)—83 (37)	0.93 (0.53, 1.63)	*0.793*	0.46 (0.19, 1.00)*
CNS	49 (68)—188 (59)	1.60 (1.16, 2.20)	0.004**	23 (32)—130 (41)	1.15 (0.75, 1.76)	*0.528*	0.68 (0.38, 1.20)
GHSP	190 (49)—694 (48)	1.69 (1.43, 1.99)	1.7E-13***	201 (51)—767 (52)	1.60 (1.37, 1.86)	1.6E-09***	0.96 (0.76, 1.20)
NS	50 (71)—205 (74)	1.56 (1.15, 2.13)	0.005**	20 (29)—72 (26)	1.64 (1.00, 2.69)	0.050*	1.14 (0.60, 2.10)
RPE	270 (43)—1 143 (38)	1.44 (1.25, 1.66)	2.9E-07***	363 (57)—1 860 (62)	1.26 (1.13, 1.40)	3.6E-05***	0.83 (0.69, 0.99)*
SLE	33 (92)—60 (88)	3.29 (2.14, 5.06)	5.9E-08***	3 (8)—8 (12)	2.64 (0.68,10.2)	*0.160*	0.68 (0.11, 3.10)
SS	105 (55)—163 (52)	3.96 (3.08, 5.1)	1.1E-26***	85 (45)—153 (48)	3.48 (2.69, 4.51)	3.4E-21***	0.86 (0.59, 1.26)
TIN	865 (63)—4 558 (65)	1.15 (1.07, 1.24)	2.0E-04***	509 (37)—2 468 (35)	1.27 (1.16, 1.4)	3.0E-07***	1.09 (0.96, 1.23)
UNN	98 (62)—405 (65)	1.47 (1.17, 1.84)	9.2E-04***	59 (38)—219 (35)	1.74 (1.32, 2.30)	8.8E-05***	1.11 (0.76, 1.62)
WG	51 (51)—132 (37)	2.54 (1.8, 3.56)	8.2E-08***	49 (49)—223 (63)	1.47 (1.09, 1.99)	0.011*	0.57 (0.35, 0.91)*

*AHA* Acquired hemolytic anemia, *ANS* Acute nephritic syndrome, *RPNS* Rapidly progressive nephritic syndrome, *CNS* Chronic nephritic syndrome, *GHSP* Glomerulonephritis due to Henoch-Schönlein purpura, *NS* Nephrotic syndrome, *RPE* Recurrent and persistent hematuria, *SLE* Systemic lupus erythematosus, *SS* Sjögren’s syndrome, *TIN* Tubulointerstitial nephritis, *UNN* Unspecific nephritis and nephropathy, *WG* Wegener’s granulomatosis

^a^Percent individuals of either CD or RI diagnosed with IMRDs either before or after the diagnosis with CD

^*^nominal *p* value < 0.05, ** nominal *p* value < 0.01, *** nominal *p* value < 0.001

**Fig. 1:**
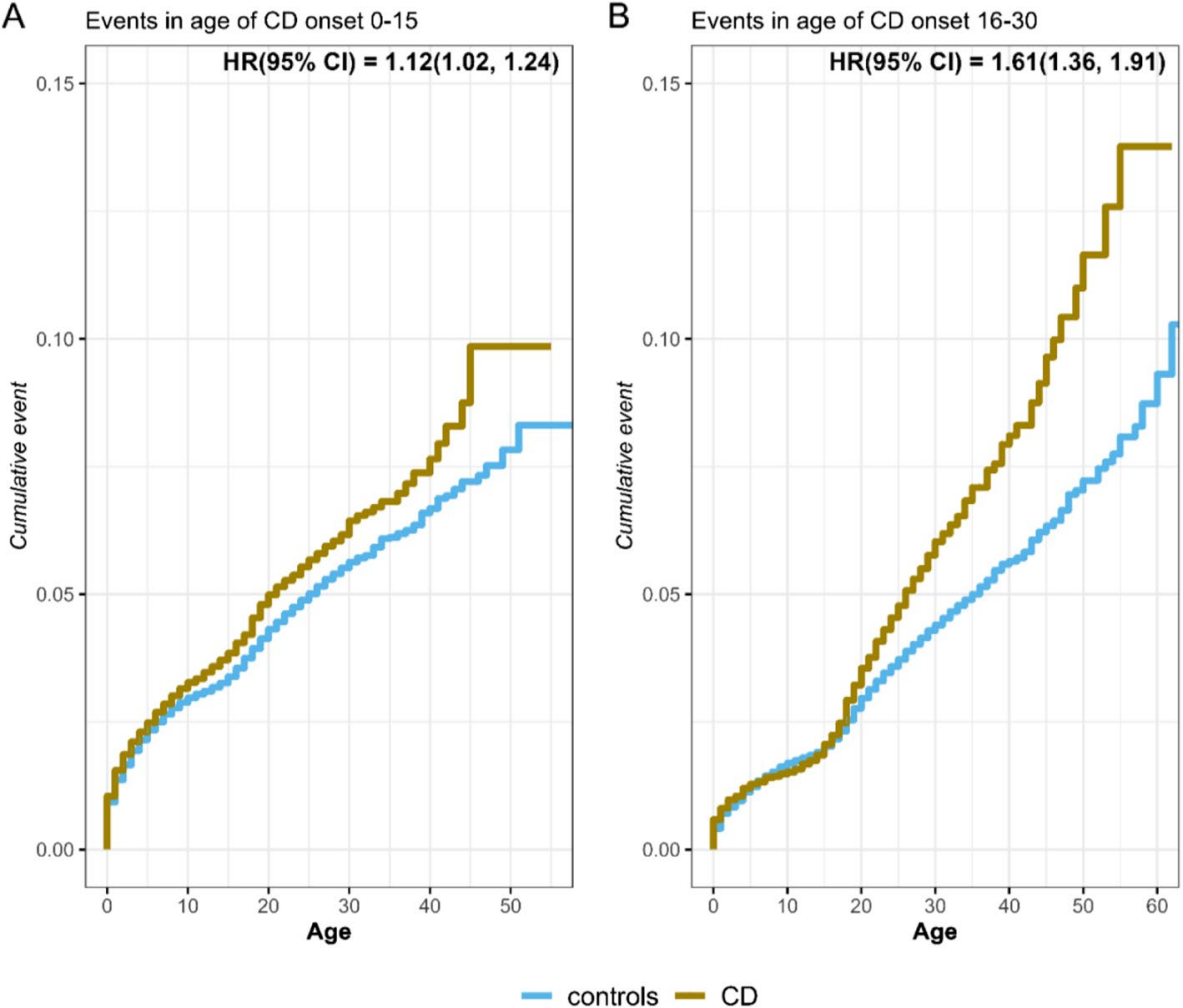
Risk of all IMRDs in CD patients and RI by age at CD diagnosis. Cumulative events of all IMRDs in different groups of age at diagnosis of coeliac disease (CD) compared with matched controls born the same year (*n* = 1:6). In **A**, cumulative events of IMRDs when the diagnosis of CD was made between the ages of 0–15. In **B**, cumulative events of IMRDs when the diagnosis of CD was made between the ages of 16–30. For CD patients diagnosed between 0 and 15 years, HR was 1.12 indicating a lower risk for CD patients diagnosed early in life compared with an HR of 1.61 in those diagnosed with CD between the ages of 16–30. This difference in the age groups was significantly different (*p* = 0.005)

We next focused on different age groups at CD diagnosis. In our main cohort, we found that most CD patients were born between 1985 and 2017 and hence diagnosed with CD before 30 years old. Thus, we analyzed the risk of developing IMRDs across subgroups of age at CD diagnosis in patients diagnosed with CD between 0 and 30 years of age. Our data indicated that CD patients were more likely to develop any IMRD if diagnosed between 16–30 years of age when compared with 0–15 years of age, as shown in Fig. 1 (HR: 1.61; *p* < 0.001 and HR: 1.12; *p* < 0.02 respectively). A comparison between the two age groups using conditional logistic regression gave an estimate of 1.16 for the interaction between CD and these two age groups in predicting risk of IMRD (*p* = 0.005). We also divided all patients into four subgroups of age at CD diagnosis: 0–10, 11–25, 26–45 and 46–75. The lowest risk was consistently found in the age group 0–10 for all IMRDs (Table S1).

## Discussion

Coeliac disease (CD) is an autoimmune disease with a long period of non-classic signs or no symptoms at all, which can cause a delay in CD diagnosis [27]. The association between unrecognized CD and the development of immune-mediated renal diseases (IMRDs) has not been investigated. Here, we hypothesized that early CD diagnosis reduces the risk of developing CD-associated IMRDs because of early dietary treatment. We also expected to find that longer duration of persistent exposure to gluten increases the risk of developing IMRDs.

Our initial analysis revealed associations between CD and ten of the twelve IMRDs investigated in this study. While some of the associations we found were reported in the literature [28–32], none of these previous studies focused on further investigating whether the risk of co-morbidity events within the CD population could be dependent on the age of CD diagnosis and thus the time of exposure to gluten. The lack of association that we observed between CD and acute nephritic syndrome (ANS) and rapidly progressive nephritic syndrome (RPNS) could simply be the result of a smaller patient population regarding these IMRDs and therefore not enough power or effect to reach significance.

Following stratification of CD participants into two subcohorts by years either before or after CD diagnosis, we observed a reduced or a similar risk following CD diagnosis for most of the IMRDs. A significantly reduced risk was found for Acute nephritic syndrome (ANS), Rapidly progressive nephritic syndrome (RPNS), Recurrent and persistent hematuria (RPE) and for Wegener’s granulomatosis (WG). The HR after a CD diagnosis was slightly increased for Nephrotic syndrome (NS), Tubulointerstitial nephritis (TIN) and Unspecific nephritis and nephropathy (UNN), however the increase was not significant in any of these three IMRDs.

In our main cohort, we found that most CD patients were born between 1985 and 2017 and hence diagnosed with CD before 30 years old. This phenomenon could be attributed to several historic events. For example, detection of auto-antibodies directed against endomysium (IgA-EMA) was routinely introduced in Sweden from the mid-1980s, as changes in infant feeding may have contributed to the epidemic pattern of CD that occurred in Sweden between 1985 and 1996 [33]. By selecting those CD patients who were diagnosed between the ages of 0 and 30, we possibly minimized confounding effects that were beyond our control and associated with changes in environmental factors occurring over time (generations) in the Swedish population.

The analysis of age at CD diagnosis revealed that IMRDs were at lower risk to develop in people with CD when a CD diagnosis occurred before the age of 15 years old compared with the age between 16–30 years old. This suggests a potential protective effect from early adoption of a gluten-free diet in the context of IMRD events. In support to these data, it has been shown that allergy to foods can be a factor inducing or triggering relapses in reported cases of pediatric Nephrotic syndrome. For example, albuminuria reduced in children with Nephrotic syndrome following withdrawal of cow’s milk and gluten products [34, 35].

The strengths of this study were the large number of individuals included from the Swedish population and the high coverage of the national registries. Although not significantly different in all, the HR was reduced in eight IMRDs following a diagnosis of CD, and the lowest risk of renal disease was found if a CD diagnosis occurred before the age of 10 for all twelve IMRDs. These data highlight the possibility that adopting a gluten-free diet early in life could affect disease.

In addition to emphasize the need to confirm a CD diagnosis as soon as possible in life in people with suspected signs of gluten intolerance and / or familiar history of autoimmune diseases at the systemic or renal level, our data suggest that diverse autoimmune phenomena associated with CD occur within the kidney. This could be due to the different immunological response in T-cells after long-standing inflammation as well as the cross-reactivity-mediated expansion on collagens that is discussed in the background section of this article. Therefore, our findings may contribute to a better understanding of the etiology of IMRDs in future studies.

A limitation of our analyses is that we had no data on dietary treatment, that is, we had to assume that all CD participants strictly adhered to a gluten-free diet following CD diagnosis. It is possible that some CD patients discontinued dietary treatment at some point during follow up potentially affecting our results. In addition, we found disease subclassification to be less accurate using ICD 9, 8 or 7 codes, and some overlap can exist in different diseases. We did not select patients specifically with renal involvement in those with Systemic lupus erythematosus, Wegener’s granulomatosis nor Sjögren’s syndrome, resulting in the inclusion of patients also without renal involvement. An additional limitation to this study is the significant trend that CD and renal disease were diagnosed around the same time in life. This could suggest that individuals with either of the two types of disease are more prone to visit a doctor compared to individuals who never get a diagnosis. Symptoms caused by CD could perhaps lead to a higher number of visits to a physician which enables earlier diagnosis of renal disease.

## Conclusions

This work identifies a reduced risk of developing immune-mediated renal diseases after a diagnosis of CD is made compared with before as well as by an early versus late age of CD diagnosis. Our data underline the value of adhering to a gluten-free diet following CD diagnosis.

## Supplementary Information

Additional file 1: Figure S1.

Additional file 2: Table S1.

## Data Availability

Data will be made available upon reasonable request to the corresponding author.

## Abbreviations

CD: Coeliac disease
IMRDs: Immune-mediated renal diseases
Ttg: Tissue transglutaminase
ICD: International classifications of Disease
HR: Hazard ratio
RI: Reference individuals
AHA: Acquired hemolytic anemia
ANS: Acute nephritic syndrome
RPNS: Rapidly progressive nephritic syndrome
CNS: Chronic nephritic syndrome
GHSP: Glomerulonephritis due to Henoch-Schönlein purpura
NS: Nephrotic syndrome
RPE: Recurrent and persistent hematuria
SLE: Systemic lupus erythematosus
SS: Sjögren’s syndrome
TIN: Tubulointerstitial nephritis
UNN: Unspecific nephritis and nephropathy
WG: Wegener’s granulomatosis
