# Supplementary material for: Unraveling the role of early coeliac disease diagnosis in the risk of developing immune-mediated renal diseases
Source: BMC Gastroenterol. 2025 Mar 3;25:125. doi: 10.1186/s12876-025-03705-5 (PMC11874109; doi:10.1186/s12876-025-03705-5)

Supplementary Figure S1. Cumulative events of all immune-mediated renal diseases (IMRDs) and of those with a significant difference in risk (hazard ratio, HR) before or the same age as a diagnosis of coeliac disease (CD) versus after, compared with matched controls born the same year. (n= 1:6)
Acute nephritic syndrome (ANS), Rapidly progressive nephritic syndrome (RPNS), Recurrent and persistent hematuria (RPE), Wegener's granulomatosis (WG).

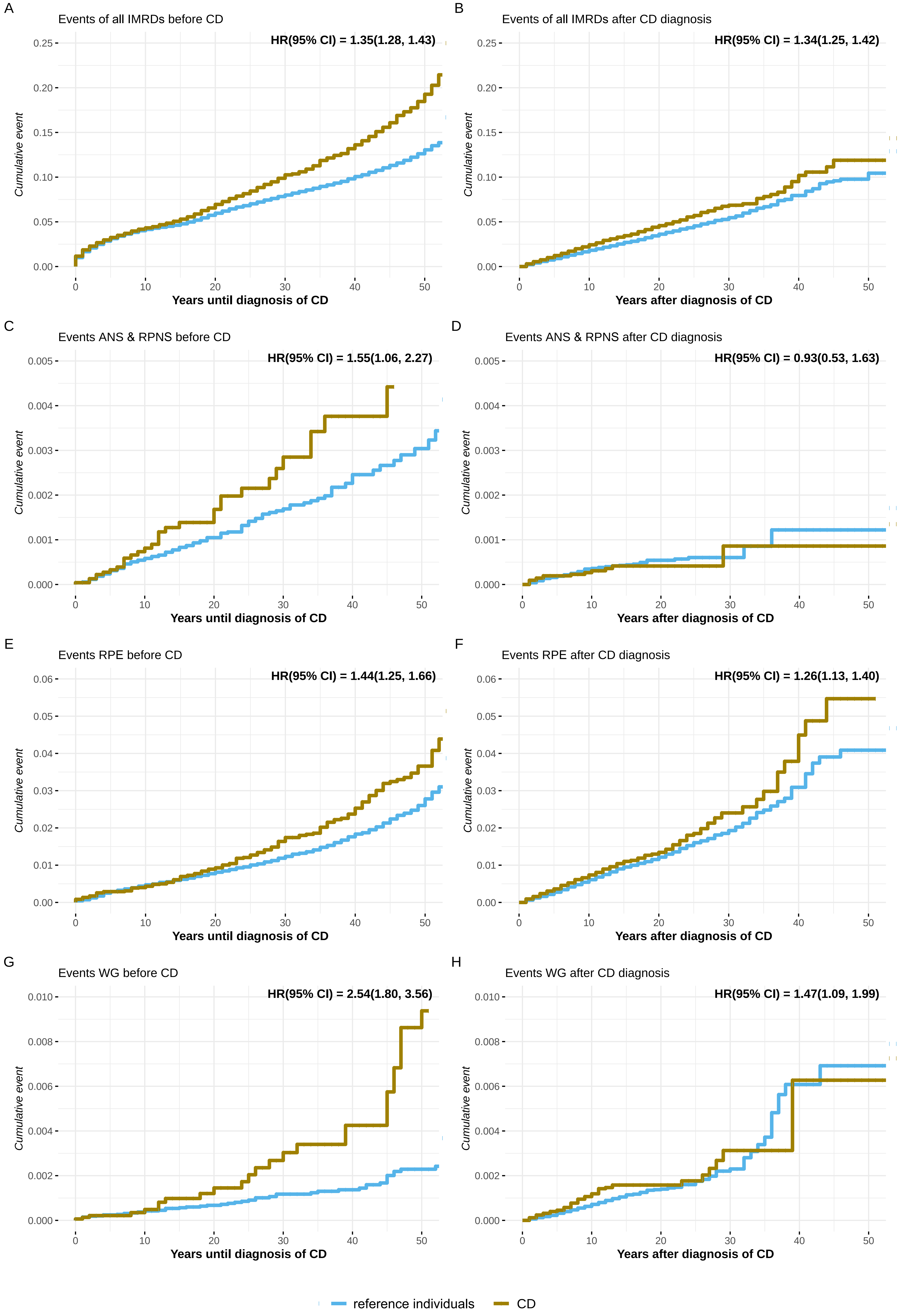

Supplement: Supplementary file 1 — Additional file 1: Figure S1. [file 12876_2025_3705_MOESM1_ESM.pdf]
