# Supplementary material for: Unraveling the role of early coeliac disease diagnosis in the risk of developing immune-mediated renal diseases
Source: BMC Gastroenterol. 2025 Mar 3;25:125. doi: 10.1186/s12876-025-03705-5 (PMC11874109; doi:10.1186/s12876-025-03705-5)
Supplement: Supplementary file 2 — Additional file 2: Table S1. [file 12876_2025_3705_MOESM2_ESM.pdf]

Table S1. Risk of immune-mediated renal diseases (IMRDs) depending on different groups of age at diagnosis of coeliac disease (CD).

Disease (ICD10)	Age of CD	HR (95 % CI)	*p-value*
**ALL IMRDS**	0-10 yrs	1.11(1.00, 1.24)	*0.05*
	11-25 yrs	1.31(1.13, 1.52)	*2.95E-04*
	26-45 yrs	1.75(1.50, 2.03)	*9.31E-13*
	46-75 yrs	1.48(1.32, 1.66)	*3.68E-11*
**AHA (D59)**	0-10 yrs	1.92(0.87, 4.26)	*0.11*
	11-25 yrs	2.15(0.78, 5.98)	*0.14*
	26-45 yrs	2.77(1.05, 7.29)	*0.04*
	46-75 yrs	4.08(2.09, 7.97)	*3.70E-05*
**ANS (N00)**	0-10 yrs	1.07(0.50, 2.26)	*0.87*
	11-25 yrs	0(0, Inf)	*1.00*
	26-45 yrs	0(0, Inf)	*1.00*
	46-75 yrs	6.02(0.38, 96.25)	*0.20*
**ANS & RPNS (N01)**	0-10 yrs	0.98(0.49, 1.99)	*0.96*
	11-25 yrs	0.32(0.04, 2.37)	*0.26*
	26-45 yrs	0.83(0.10, 6.64)	*0.86*
	46-75 yrs	1.94(0.53, 7.06)	*0.32*
**CNS (N03)**	0-10 yrs	0.51(0.18, 1.41)	*0.19*
	11-25 yrs	1.36(0.60, 3.08)	*0.46*
	26-45 yrs	0.94(0.33, 2.69)	*0.91*
	46-75 yrs	2.27(1.10, 4.67)	*0.03*
**GHSP (D69)**	0-10 yrs	1.29(1.02, 1.62)	*0.03*
	11-25 yrs	1.64(1.12, 2.41)	*0.01*
	26-45 yrs	2.54(1.70, 3.82)	*6.44E-06*
	46-75 yrs	1.86(1.38, 2.51)	*4.18E-05*
**NS (N04)**	0-10 yrs	1.33(0.62, 2.87)	*0.46*
	11-25 yrs	2.39(0.75, 7.63)	*0.14*
	26-45 yrs	1.59(0.45, 5.65)	*0.47*
	46-75 yrs	1.88(0.70, 5.09)	*0.21*
**RPE (N02, R31)**	0-10 yrs	0.97(0.76, 1.23)	*0.78*
	11-25 yrs	1.34(1.01, 1.79)	*0.04*
	26-45 yrs	1.83(1.46, 2.28)	*9.80E-08*
	46-75 yrs	1.17(0.99, 1.39)	*0.07*
**SLE (M32.14)**	0-10 yrs	0(0, Inf)	*1.00*
	11-25 yrs	0(0, Inf)	*1.00*
	26-45 yrs	3.22(0.29, 35.54)	*0.34*
	46-75 yrs	19.93(2.06, 192.41)	*0.01*
**SS (M35.0)**	0-10 yrs	2.17(1.15, 4.10)	*0.02*
	11-25 yrs	2.75(1.62, 4.67)	*1.76E-04*
	26-45 yrs	3.69(2.27, 6.01)	*1.45E-07*
	46-75 yrs	5.51(3.40, 8.92)	*3.73E-12*
**TIN (N10-12)**	0-10 yrs	1.13(0.98, 1.30)	*0.10*
	11-25 yrs	1.25(1.03, 1.51)	*0.02*
	26-45 yrs	1.38(1.06, 1.80)	*0.02*
	46-75 yrs	1.62(1.33, 1.98)	*1.37E-06*

**UNN (N05, N06 & N08)**	0-10 yrs	1.13(0.69, 1.84)	*0.63*
	11-25 yrs	2.27(1.23, 4.20)	*0.01*
	26-45 yrs	1.16(0.48, 2.77)	*0.74*
	46-75 yrs	2.88(1.80, 4.59)	*9.59E-06*
**WG (M31)**	0-10 yrs	0.57(0.13, 2.44)	*0.45*
	11-25 yrs	1.41(0.48, 4.20)	*0.53*
	26-45 yrs	1.49(0.77, 2.88)	*0.24*
	46-75 yrs	1.62(1.12, 2.33)	*0.01*

Acquired hemolytic anemia ***(AHA)***, Acute nephritic syndrome ***(ANS)***, Rapidly progressive nephritic syndrome ***(RPNS)***, Chronic nephritic syndrome ***(CNS)***, Glomerulonephritis due to Henoch-Schönlein purpura ***(GHSP)***, Nephrotic syndrome ***(NS)***, Recurrent and persistent hematuria ***(RPE)***, Systemic lupus erythematosus ***(SLE)***, Sjögren's syndrome ***(SS)***, Tubulointerstitial nephritis ***(TIN)***, Unspecific nephritis and nephropathy ***(UNN)***, Wegener's granulomatosis ***(WG)***.
